# Comprehensive Two-Dimensional Pediatric Echocardiographic Nomograms for Coronary Artery Sizes in Caucasian Children and Comparison among Major Nomograms

**DOI:** 10.3390/diagnostics14101029

**Published:** 2024-05-16

**Authors:** Massimiliano Cantinotti, Marco Scalese, Francesca Valeria Contini, Eliana Franchi, Cecilia Viacava, Giulia Corana, Alessandra Pizzuto, Marchese Pietro, Giuseppe Santoro, Nadia Assanta

**Affiliations:** 1 Fondazione G. Monasterio CNR-Regione Toscana, Via Aurelia Sud, 54100 Massa, Italy; franchi@ftgm.it (E.F.); cecilia.viacava@ftgm.it (C.V.); corana@ftgm.it (G.C.); apizzuto@ftgm.it (A.P.); pietro@ftgm.it (M.P.); gsantoro@ftgm.it (G.S.); assanta@ftgm.it (N.A.); 2 Fondazione G. Monasterio CNR-Regione Toscana, Via Giuseppe Moruzzi, 1, 56124 Pisa, Italy; 3 Department of Statistics, National Research Institute, National Research Council, 56121 Pisa, Italy; scalese@ifc.cnr.it; 4 Clinical Cardiology Unit, University of Cagliari, 09042 Monserrato, Italy; fvcontini@gmail.com

**Keywords:** coronary arteries, normal values, echocardiography, children

## Abstract

Background: Although coronary artery nomograms in children have been published, data on Caucasian children are lacking. The aim of this study is to provide: (i) a full dataset of coronary artery diameters in healthy children and (ii) a comparison among major previous nomograms. Materials and Methods: We prospectively evaluated 606 healthy subjects (age range, 1 days–<18 years; median age 8.7 years; 62.5% male). Coronary artery measurements in a short-axis view were performed. Age, heart rate, and body surface area (BSA) were used as independent variables in different analyses to predict the mean values of each measurement. To assess the accuracy of the predictive models of different studies, a Z-score calculator was created using Lopez’s nomograms for comparison. Results: The association with BSA was found to be stronger, and was used for normalization of our data. The best-fit models, satisfying the assumption of homoscedasticity and normality of residuals and showing the highest R^2^ scores, were logarithmic (ln[y] = a + b*ln[x]). Predicted values and Z-score boundaries by BSA are provided. Our ranges of normality are slightly lower than those, diverging from −0.22 to −0.59 Z-scores for the left main coronary artery and from −0.23 to −0.3 Z-scores for the right coronary artery. Conclusions: We report a complete dataset of normal echocardiography coronary artery diameter (including new measures of the proximal origin) values in a large population of healthy children. Our data were statistically like those of north American nomograms.

## 1. Background

Pediatric echocardiography coronary artery nomograms have been proposed by different authors and in different ethnicities [1,2,3,4,5,6,7]. The most recent nomograms were calculated by using a large dataset, enrolling from 506 [4] to 3215 [3] healthy children (0–18 years). Data on Caucasian children, however, are lacking. Not all the measurements are reported by all the nomograms, and some data are missing. For instance, data on circumflex arteries (CX) [1,2,4] are relatively limited. Measurement of the coronary artery, furthermore, has been uniformly performed by different authors [1,2,3,4,5,6,7,8] in short-axis view, 3 to 5 mm distal to their origin. Despite this, measurement of the left main coronary artery (LMCA) and the right coronary artery (RCA) origin is often performed more proximally immediately at their origin [9,10]. A proximal dilatation of the LMCA and RCA has also been described in children after COVID-19 infection [9,10]. Thus, the availability of normal pediatric data on proximal LMCA and RCA diameters, which are currently lacking, may be helpful in specific clinical settings. Data on LMCA’s origin and bifurcation distance are also lacking. 

A comparison among current Z-score sources [1,2,3,4,5,6,7] has never been performed so far, and differences among them have not been underscored. Whether differences among the nomogram sources utilized may have clinical implications in under- or overestimation of disease severity, as well as which Z-score source should be employed in clinical practice, are also unknown. 

The aim of the present investigation was to provide a complete dataset of coronary artery measurements in a large population of healthy Caucasian children, including new measurements (e.g., LMCA and RCA proximal origin, LMCA origin to bifurcation distance). Another aim is to provide a comparison among major previous datasets of normal values. 

## 2. Material and Methods

### 2.1. Study Population

Healthy Caucasian children were prospectively recruited at the Fondazione CNR-Regione Toscana G. Monasterio of Massa from July 2021 to November 2022. Exclusion criteria have been previously reported [8]. All subjects with clinical, electrocardiographic, or echocardiographic evidence of congenital heart disease (CHD) or acquired heart disease were excluded. Other exclusion criteria consisted of patients with known or suspected neuromuscular disease, genetic syndromes, or chromosomal abnormalities; body mass index ≥ 95th percentile for children ≥ 2 years old or weight-for-length Z-scores ≥ 2; pulmonary hypertension; systemic hypertension (for children > 4 year of age); connective tissue disorder; or family history of genetic cardiac disease. All children with recent (e.g., ≤3 months previous the examination) histories of severe COVID-19 infection were excluded from the study. The study was approved by the local ethics committee (Study ‘‘Bet’’ No. 390). Parents or legal guardians were informed and agreed to participate by providing written consent.

### 2.2. Echocardiographic Measurements

A complete echocardiographic examination was performed for all the subjects at a frame rate of 60 to 100 frames/s using a Vivid E95 system (GE Ultrasound, GE HealthCare, Chicago, IL, USA). Two experienced pediatric cardiologists (M.C., E.F.) acquired the images, and two other experienced operators (P.M., N.A.) performed off-line analysis on a dedicated workstation (Echopac V.202, GE Healthcare). 

**Figure 1:**
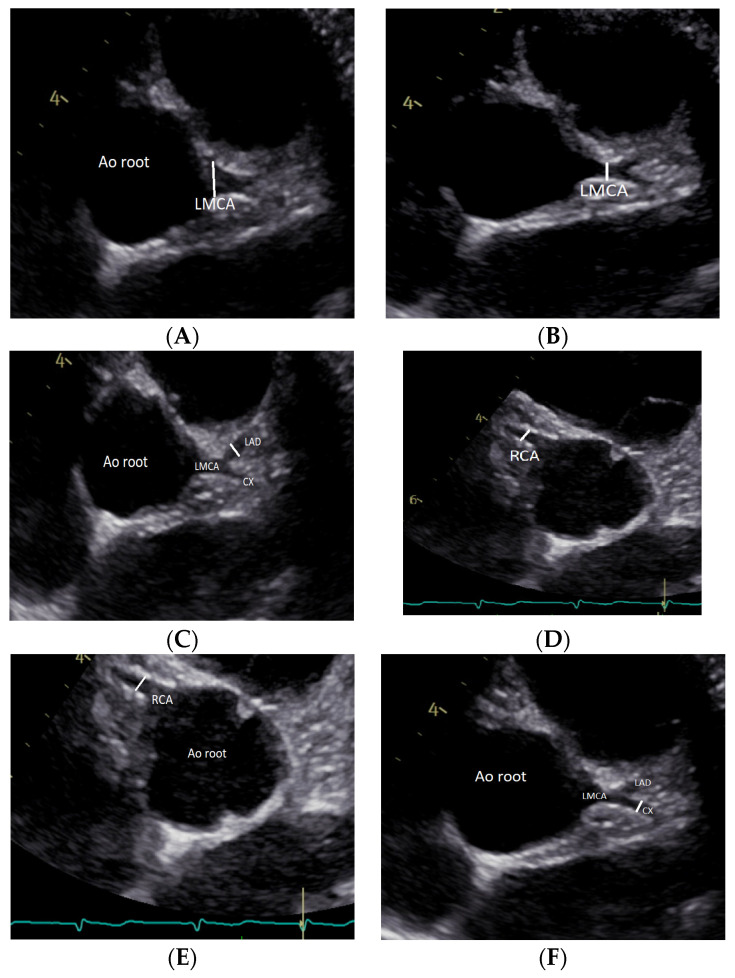
(**A**) Left main coronary artery (LMCA) at its proximal origin; (**B**) LMCA at 3–5 mm from its origin; (**C**) left anterior descending artery (LAD); (**D**) right coronary artery (RCA) at its proximal origin, (**E**) LMCA at 3–5 mm from its origin; (**F**) circumflex artery.

The diameters of the RCA, LAD, and CX were measured at 3 to 5 mm distal to their origins in the parasternal short-axis view. The diameter of the LMCA was measured at the midpoint between the ostium of the LMCA and the bifurcation of the LAD and CX in the parasternal short-axis view. The diameters of LMCA and RCA were also calculated at their origin from the aortic root (Figure 1). The distance between the LMCA coronary artery origin and its bifurcation into LAD and CX was also calculated in the short-axis view. For any given parameter, measurements were only made if excellent and unambiguous views were available. Measurements were repeated three times on the same dataset, and the mean was calculated. Inter- and intra-rater agreement of measurements was based on the coefficients of variation (CVs) and intraclass correlation coefficients (ICCs) in 20 subjects. The inter- and intra-CV were calculated similarly to an average value calculated from the individual CVs for all the duplicates. Inter-CVs of less than 15% were generally acceptable, while Intra-CVs should be less than 10%. The Statistical Package for Social Sciences (SPSS) Release 23.0 (Chicago, IL, USA) and Stata Version 13 for Windows (Stata Corp, College Station, TX, USA, 2001) were used for the analyses.

### 2.3. Statistical Analysis

The sample size was calculated based on previous observations [8]. Multiple models using linear, logarithmic, exponential, and square root equations were tested to evaluate the relationship between the parameters of body size, heart rate, age, and each of the echocardiographic variables. The model with the highest R2 value was considered to provide the best fit (among those satisfying the assumption of homoscedasticity). Heteroskedasticity was tested using the White and the Breusch–Pagan tests [11], while the normality of residuals was tested with the Shapiro–Wilk and Lilliefors (Kolmogorov–Smirnov) tests [12]. The Haycock formula was used to calculate body surface area (BSA). Outliers were identified visually and by using the leverage values and the studentized error residuals; those observations were omitted from the final analysis if they significantly deviated from the models. The effects of gender were also evaluated as covariates in these models.

Rates of intra-observer and inter-observer variability were calculated from 20 randomly selected subjects. 

The predictive values and the z scores of measurements (RCA, LAD, LMCA, and CX) from each study [1,3,4] were calculated using the equations illustrated in the original manuscripts. The nomogram from Lopez et al. [3] was utilized as the “gold standard” for comparison, as it was the most recent and had the largest sample size among all nomograms assessed. In this comparison, Z-scores represent how many standard deviations (SDs) of Cantinotti, Dallaire [1], and Zhang [4] were calculated using the Lopez [3] mean and SD. 

## 3. Results

Of 640 patients initially enrolled, 34 were excluded for poor image quality (20 subjects) or incomplete acquisition (14 subjects). Accordingly, the feasibility was 94.6%. The final study population included 606 healthy subjects (age range, 1 days–<18 years; median age 8.7 years; 62.5% male), and BSA ranged from 0.17 to 2.09 m^2^ (Table 1).

### 3.1. Building of Z-Scores

**Figure 2:**
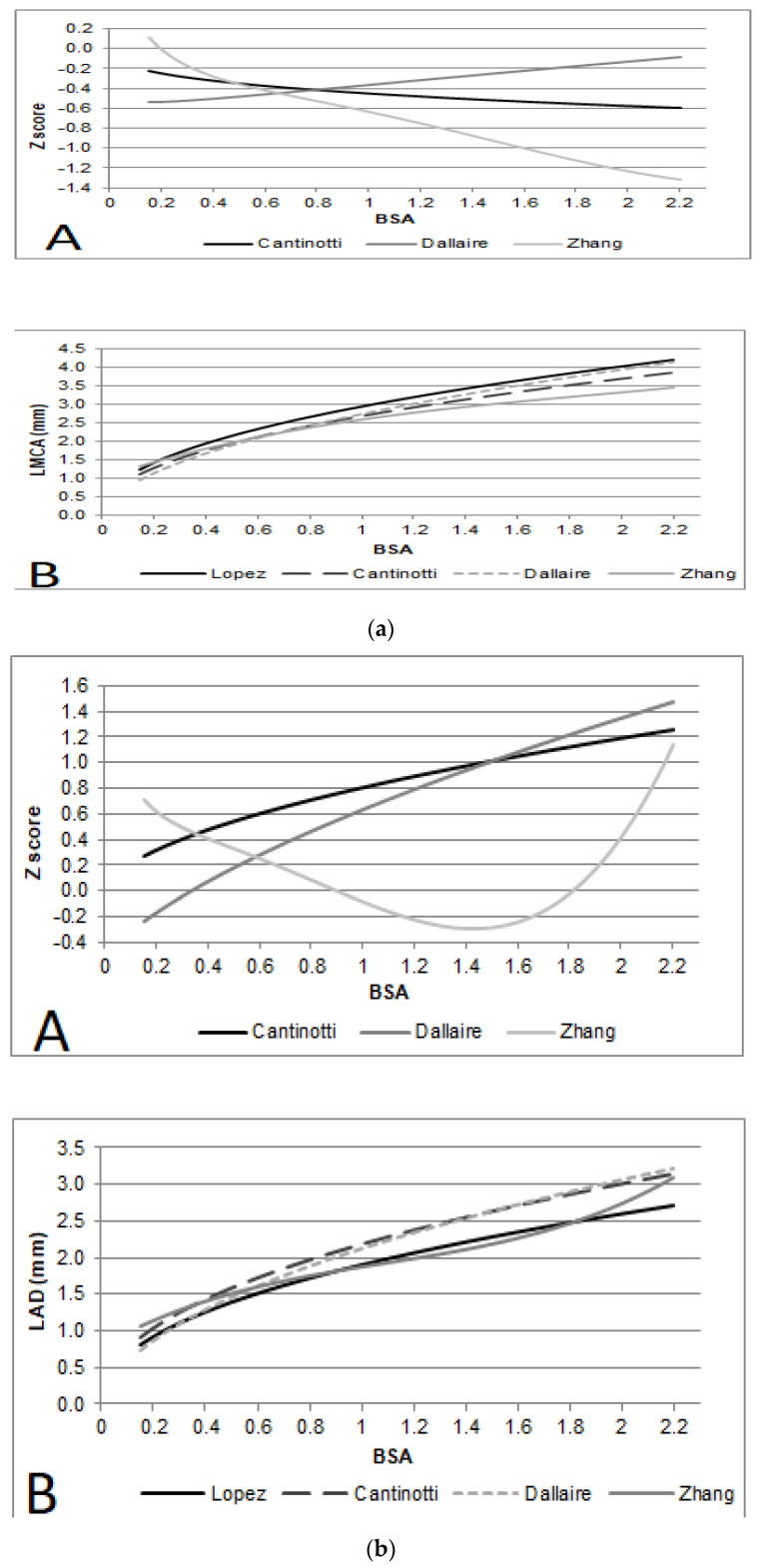

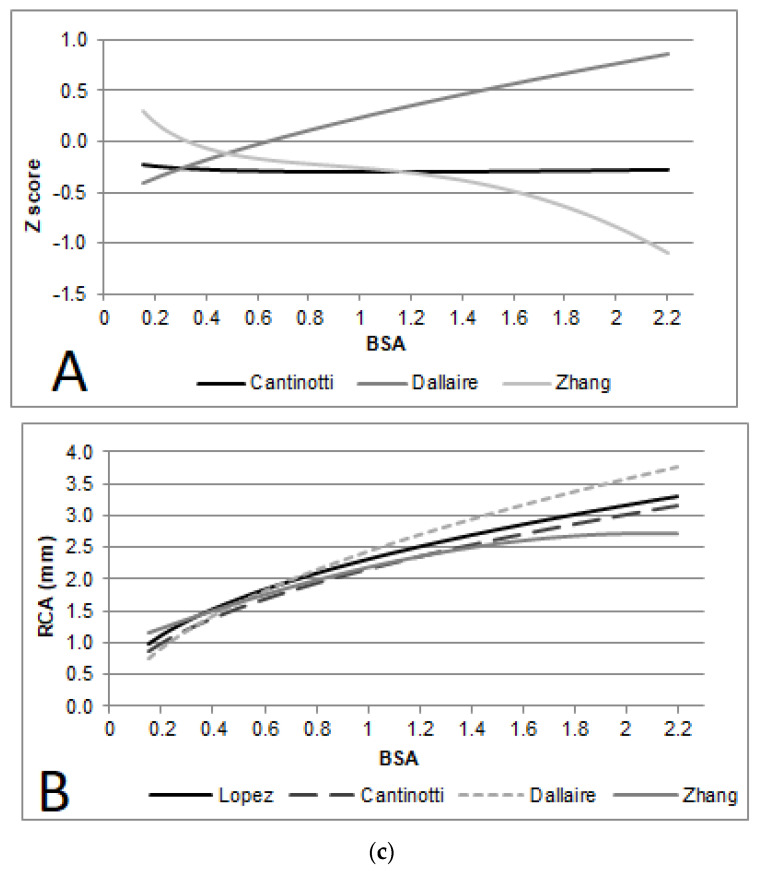
(**a**) Left main coronary artery (LMCA) (**A**) Comparison of Dallaire et al., Lopez et al., and Cantinotti et al. with Lopez’s Z-scores used as gold standard; (**B**) Z score chart by Lopez et al., Dallaire et al., Zhang et al., and Cantinotti et al. (**b**) Left anterior descending artery (LAD). (**A**) Comparison of Dallaire et al., Lopez et al., and Cantinotti et al. with Lopez’s Z-scores used as gold standard; (**B**) Z score chart by Lopez et al., Dallaire et al., Zhang et al., and Cantinotti et al. (**c**) Right coronary artery (RCA). (**A**) Comparison of Dallaire et al., Lopez et al., and Cantinotti et al. with Lopez’s Z-scores used as gold standard (**B**) Z score chart by Lopez et al., Dallaire et al., Zhang et al., and Cantinotti et al. [1,3,4].

The measurements were first modeled with HR, age, weight, height, and BSA. BSA provided the best fit. For all measurements, linear, logarithmic, exponential, and square root models were evaluated for best fit, and tests for heteroscedasticity were applied. The best-fit models, satisfying the assumption of homoscedasticity and normality of residuals and showing the highest R^2^ scores, were logarithmic (ln[y] = a + b*ln[x]) (Table 2). Z-scores for the whole population are provided in Table 3 and Figure 2.

### 3.2. Confounders: Gender

The influence of gender on the measured parameters were evaluated by multiple linear regression models, using gender as covariate along with BSA. A small but significant effect of gender was found in the model for all measurements. However, because the effects were not clinically relevant for all measurements, gender was not included in the final models. As detailed in Supplemental Table S1, the ICC was high for all the measurements, showing an almost perfect agreement. Furthermore, the CV was very low, confirming an excellent agreement.

### 3.3. Differences among LMCA Proximal Measurements and Those Performed at 5 mm from Its Origin

The LMCA and RCA diameter at their proximal origin were higher than those measured at 5 mm from the origin (both *p* < 0.001) (Table 3).

## 4. Comparison among Authors

A comparison among our results and three previous studies [1,3,4] with mean values are provided for a range of BSA (0.15–2.20 m^2^) for LMCA, LAD, RCA and CX (Figure 2). Z-score charts are provided for a range of BSA (0.15–2.20 m^2^) for each nomogram assessed in this study (Figure 2). Moreover, plots of Z-scores over the same range of BSA were generated to assess trends among different studies. 

### 4.1. Left Main Coronary Artery

For LMCA diameters, Lopez et al. [3] showed slightly higher values compared to others, while the values proposed by Dallaire et al. [1] were the lowest at a low BSA, and those by Zhang et al. [4] were the lowest at high BSA values. Compared to the values proposed by Lopez et al., the data of Zhang and colleagues underestimated the LMCA diameter from a BSA of 0.2 m^2^, and this difference increased as BSA increased (with a difference from 0.03 at a BSA of 0.2 up to −1.22 Z score at a BSA of 2 m^2^). For data proposed by Dallaire et al., the difference with Lopez et al. decreased as BSA increased (e.g., from −0.54 at a BSA of 0.15 m^2^ to −0.11 at a BSA of 2 m^2^). Our data are quite similar to those proposed by Lopez et al. [3], diverging from −0.22 to −0.59 Z-scores.

### 4.2. Left Anterior Descending Artery

For LAD diameters, we showed slightly higher mean values compared to Lopez et al. [3], and the difference increased as BSA increased (from +0.27 score at 0.15 m^2^ to 1.18 Z-score at a BSA of 2 m^2^). Dallaire [1] showed the lowest LAD mean values at a low BSA (with a difference from Lopez et al. of −0.24 at a BSA of 0.15 m^2^), and the highest at high BSA values (with a difference from Lopez et al. of +1.35 at a BSA of 2 m^2^). For BSA values >0.9 m^2^, the predicted LAD mean values we proposed are practically the same as those of Dallaire et al. [1]. The predicted LAD mean values provided by Zhang et al. [4] are slightly higher than those of Lopez et al. up to a BSA of 0.8 m2 (from +0.71 Z-score at a BSA of 0.15 m^2^ to +0.039 Z-score at a BSA 0.85 m^2^), and vert was similar to Lopez et al. [3] from a BSA of 0.8 m^2^ to a BSA of 1.8 (Z-scores differences ranging from −0.001 to −0.24).

### 4.3. Right Coronary Artery

The predicted values of RCA diameters that we proposed are only slightly slower than those of Lopez et al. [3] (with differences of −0.23 to –0.3 Z-scores). For values of BSA greater than 0.5 m^2^, the predicted values of Zhang et al. [4] were the lowest, with differences from Lopez et al. ranging from −0.46 to 1.1 Z-scores. For values of BSA greater than 0.85 m^2^, the predicted values of Dallaire et al. [1] were the highest, with differences from Lopez et al. [3] ranging from +0.11 up to +0.85 Z-scores. 

### 4.4. Circumflex Artery

Lopez’s nomograms [3] do not report data for circumflex arteries; thus, comparison was not feasible in this case. Compared to Dallaire nomograms [1], the mean values of CX diameters that we proposed were higher for BSA < 0.4 m^2^, equal at BSA = 0.4 and lower for BSA greater than 0.4 m^2^ (and the difference increases as BSA increases). Comparing all the nomograms for values of BSA greater than 0.45 m^2^, the predicted values of Zhang et al. [4] were the lowest.

## 5. Discussion

This is the first study to provide a complete dataset of measurements of coronary arteries in a homogenous population of Caucasian children. As expected, our data confirm a linear increase in coronary artery sizes with somatic growth [1,2,3,4]. For all the measurements modeled with heart rate, age, weight, height, and BSA were tested, and BSA provided the best fit. Thus, in accordance with previous reports [1,2,3,4], Z-scores were provided according to BSA. The normalization for BSA that we employed is also the one that is commonly employed (and recommended) for other cardiac structures in the pediatric age range [13]. Gender had a small, but not clinically relevant, effect on the models; thus, the data were not divided by gender. 

Compared to previous reports [1,2,3,4], we provide some additional measurements, including the measurements of the left main coronary artery and of the right coronary artery at their very proximal origin in short-axis view, as well as the distance among LMCA origin and its bifurcation in short-axis view. As expected, proximal diameters were significantly higher for both left main coronary artery and the right coronary artery. For example, for a given child with a given BSA of 0.80, the predicted values of the left main coronary artery diameter were 3.03 mm at its very proximal origin and 2.43 mm at 3–5 mm from its proximal origin. For the same child, the right coronary artery diameter at its proximal origin was 2.40 mm, while, using a conventional measurement, the diameter was 1.93 mm. Measurements of very proximal diameters are often performed, and data are erroneously compared with current Z-scores (using a measurement at 3–5 mm from proximal origin) [1,2,3,4], leading to an overestimation of disease severity. A dilatation of the left main coronary artery and right coronary artery limited to their proximal origin, furthermore, has been described in children affected by COVID-19 infection [9,10]. Thus, these new data may help to conduct a better examination of disease severity even when a proximal measurement is performed. Interestingly, we demonstrated not only a linear increase in coronary artery diameter with increasing BSA, but also an increase in the distance between the left main coronary artery origin and its bifurcation in a short-axis view. 

In the present investigation, we also proposed a comparison among most recent and wider nomograms (Lopez et al., Dallaire et al., Zhang et al.) [1,3,4], providing numerical and visual examples of differences among them. Differences between the range of normality that we proposed and those of Lopez et al. [3] are quite limited. For the left main coronary artery, the ranges of normality proposed by Lopez et al. [3] were the highest, diverging from −0.22 to −0.59 Z-scores from our data. As a result, for a given diameter of the left main coronary artery, the Z-scores found by Lopez et al. [3] are lower than those obtained with other sources [1,4]. These differences may have clinical relevance in the estimation of a disease, from mild dilatation to severe dilatation. For instance, for a given child with a given BSA of 0.35 m^2^ and a left main coronary artery diameter of 2.7 mm, Z-scores varied from +2.42 (Lopez) [3] to +2.75 (our data) and +3.49 (Dallaire) [1]. This difference increased with higher BSA. For instance, for a given child with a given BSA of 1 m^2^ and a left main coronary artery diameter of 4 mm, Z-scores varied from +1.84 (Lopez) [3] to +2.25 (our data) and +2.90 (Dallaire) [1]. 

Also, the right coronary artery mean values that we proposed were lower than those proposed by Lopez et al. [3], diverging from −0.23 to −0.3 Z-scores. Right coronary artery differences among various nomograms, however, had limited clinical significance. For instance, for a given child with a given BSA of 0.35 and a right coronary artery diameter of 2.5 mm, limited differences of Z-score were noted, with values ranging from +3.53 (Dallaire) [1] to +3.41 (our data) and +3.07 (Lopez) [3].

We also provided values of normality for the circumflex artery, the data on which are relatively limited in the literature [1,2,4]. The range of normality we proposed was higher than those of Zangh and colleagues [4], while differences from the Dallaire nomograms [1] were not linear. Our range of normality, in fact, was higher than those of Dallaire and colleagues [1] for BSA < 0.4 m^2^, equal at BSA = 0.4, and lower for BSA greater than 0.4 m^2^, becoming clinically significant as the BSA increased.

## 6. Strengths and Limitations

We provide a complete dataset of coronary artery diameters in children, including some innovative parameters such as LMCA, RCA, and very proximal diameter. Establishing the exact origin of the coronary artery origin may be difficult, and a clear definition on how to define coronary artery origin in a short-axis view is lacking [14]. The coronary ostium, furthermore, may have an irregular shape (oval and not exactly circular/round) [15,16]; thus, slight angulation of the probe may result in a significantly different estimation of its major diameter. Three-dimensional echocardiography may provide a better examination of the ostial area and diameters [17,18], but its application may present significant limitations for very small structures, especially at high heart rates such as those encountered in neonates, infants, and young children. Widespread utilization of 3D echocardiography for coronary artery screening is inapplicable at present.

A comparison of the data of Kobayashi and colleagues [2] was not feasible, since the authors provided different z-score equations for males and females, while no other authors [1,3] differentiated between genders.

## 7. Conclusions

A complete dataset of echocardiography coronary artery diameter (including new data such as their proximal origin and LMCA-to-bifurcation distance) normal values in a large population of healthy children has been provided. Differences among different Z-score sources have been also highlighted, with practical examples of their implication in a clinical setting. These data may serve as a baseline for children presenting with suspicion of coronary artery dilatation.

## Figures and Tables

**Table 1:** (**A**). Distribution of BSA calculated with the Haycock formula. (**B**). Distribution of age.

(A)		
BSA	*N*	%
0.20–0.30	31	5.1
0.30–0.40	22	3.6
0.40–0.50	21	3.5
0.50–0.60	23	3.8
0.60–0.70	38	6.3
0.70–0.80	45	7.4
0.80–0.90	59	9.7
0.90–1.00	45	7.4
1.00–1.10	53	8.7
1.10–1.20	46	7.6
1.20–1.30	45	7.4
1.30–1.40	40	6.6
1.40–1.50	44	7.3
1.50–1.60	31	5.1
1.60–1.70	24	4.0
≥1.70	39	6.4
Total	606	100
**(B)**		
Age	*N*	%
0 days–12 months	66	11.1
1–2 years	12	2.0
2–5 years	76	12.5
5–11 years	251	41.4
11–18 years	200	33.0
Total	606	100.0

BSA = body surface area.

**Table 2:** Coefficients for regression equations, showing echocardiographic measurements and body surface area, the Standard Error of the Estimate, and the determination coefficient. Normality test: Shapiro–Wilk and Lilliefors (Kolmogorov–Smirnov). Heteroscedasticity test (White test and Breusch–Pagan test). BSA HAYCOCK. (ln[y] = a + b*ln[x]); Z value = (ln[Measurement] − (Intercept + B*ln[BSA]))/√MSE.

Measurement	Intercept	B	SEE (√MSE)	R^2^	SW	KS	BP	W
LMCA at prox. orig.	1.217	0.486	0.187	0.628	0.290	0.099	0.052	0.027
LMCA 2	0.991	0.459	0.176	0.629	0.086	0.083	0.066	0.117
LAD	0.776	0.465	0.156	0.689	0.046	0.079	0.035	0.136
CX	0.616	0.466	0.164	0.668	0.005	0.071	0.369	0.556
RCA at prox. orig.	0.985	0.492	0.188	0.627	0.714	0.200	0.684	0.913
RCA 2	0.767	0.484	0.193	0.607	0.012	0.051	0.886	0.893
LMCA prox. orig-bifurcation	1.596	0.537	0.342	0.388	0.124	0.073	0.811	0.089

LMCA = left main coronary artery; prox. orig. = proximal origin, LAD = left anterior descending artery, CX = circumflex, RCA = right coronary artery; BP, Breusch–Pagan test; KS, Kolmogorov–Smirnov test; MSE, mean square error; R^2^, coefficient of determination; SEE, standard error of the estimate; SW, Shapiro–Wilk test; W, White test.

**Table 3:** Predicted values (mean ± 2SD) of measured echocardiography variables expressed by body surface area (BSA) (Haycock).

BSA	**0.15**	**0.20**	**0.25**	**0.30**	**0.35**	**0.40**	**0.50**	**0.60**	**0.70**	**0.80**	**0.90**
	0.92	1.06	1.18	1.29	1.39	1.49	1.66	1.81	1.95	2.08	2.21
LMCA at prox. orig.	**1.34**	**1.54**	**1.72**	**1.88**	**2.03**	**2.16**	**2.41**	**2.63**	**2.84**	**3.03**	**3.21**
	1.95	2.25	2.50	2.73	2.95	3.14	3.50	3.83	4.13	4.40	4.66
	0.79	0.91	1.00	1.09	1.17	1.24	1.38	1.50	1.61	1.71	1.81
LMCA	**1.13**	**1.29**	**1.43**	**1.55**	**1.66**	**1.77**	**1.96**	**2.13**	**2.29**	**2.43**	**2.57**
	1.60	1.83	2.03	2.20	2.37	2.52	2.79	3.03	3.25	3.46	3.65
	0.66	0.75	0.83	0.91	0.98	1.04	1.15	1.25	1.35	1.43	1.51
LAD	**0.90**	**1.03**	**1.14**	**1.24**	**1.33**	**1.42**	**1.57**	**1.71**	**1.84**	**1.96**	**2.07**
	1.23	1.40	1.56	1.70	1.82	1.94	2.15	2.34	2.51	2.68	2.83
	0.55	0.63	0.70	0.76	0.82	0.87	0.97	1.05	1.13	1.20	1.27
CX	**0.76**	**0.87**	**0.97**	**1.06**	**1.14**	**1.21**	**1.34**	**1.46**	**1.57**	**1.67**	**1.76**
	1.06	1.21	1.35	1.47	1.58	1.68	1.86	2.03	2.18	2.32	2.45
	0.72	0.83	0.93	1.02	1.10	1.17	1.31	1.43	1.54	1.65	1.75
RCA at prox. orig.	**1.05**	**1.21**	**1.35**	**1.48**	**1.60**	**1.71**	**1.90**	**2.08**	**2.25**	**2.40**	**2.54**
	1.53	1.77	1.97	2.16	2.33	2.48	2.77	3.03	3.27	3.49	3.70
	0.58	0.67	0.75	0.82	0.88	0.94	1.05	1.14	1.23	1.31	1.39
RCA	**0.86**	**0.99**	**1.10**	**1.20**	**1.30**	**1.38**	**1.54**	**1.68**	**1.81**	**1.93**	**2.05**
	1.26	1.45	1.62	1.77	1.91	2.03	2.26	2.47	2.67	2.84	3.01
	0.90	1.05	1.18	1.30	1.42	1.52	1.72	1.89	2.06	2.21	2.35
LMCA prox. orig.-bifurcation	**1.78**	**2.08**	**2.34**	**2.58**	**2.81**	**3.02**	**3.40**	**3.75**	**4.07**	**4.38**	**4.66**
	3.53	4.12	4.64	5.12	5.56	5.98	6.74	7.43	8.07	8.67	9.24
BSA	**1.0**	**1.1**	**1.2**	**1.3**	**1.4**	**1.5**	**1.6**	**1.7**	**1.8**	**1.9**	**2.0**
	2.32	2.43	2.54	2.64	2.74	2.83	2.92	3.01	3.09	3.17	3.25
LMCA 1	**3.38**	**3.54**	**3.69**	**3.84**	**3.98**	**4.11**	**4.24**	**4.37**	**4.49**	**4.61**	**4.73**
	4.91	5.14	5.36	5.58	5.78	5.98	6.17	6.35	6.53	6.71	6.87
	1.89	1.98	2.06	2.14	2.21	2.28	2.35	2.42	2.48	2.54	2.60
LMCA 2	**2.69**	**2.81**	**2.93**	**3.04**	**3.14**	**3.24**	**3.34**	**3.44**	**3.53**	**3.62**	**3.70**
	3.83	4.00	4.16	4.32	4.47	4.61	4.75	4.89	5.02	5.14	5.27
	1.59	1.66	1.73	1.80	1.86	1.92	1.98	2.04	2.09	2.14	2.20
LAD	**2.17**	**2.27**	**2.37**	**2.45**	**2.54**	**2.62**	**2.70**	**2.78**	**2.86**	**2.93**	**3.00**
	2.97	3.10	3.23	3.35	3.47	3.58	3.69	3.80	3.90	4.00	4.10
	1.33	1.39	1.45	1.51	1.56	1.61	1.66	1.71	1.75	1.80	1.84
CX	**1.85**	**1.94**	**2.02**	**2.09**	**2.17**	**2.24**	**2.30**	**2.37**	**2.43**	**2.50**	**2.56**
	2.57	2.69	2.80	2.90	3.01	3.10	3.20	3.29	3.38	3.47	3.55
	1.84	1.93	2.01	2.09	2.17	2.24	2.32	2.39	2.46	2.52	2.59
RCA 1	**2.68**	**2.81**	**2.93**	**3.05**	**3.16**	**3.27**	**3.37**	**3.48**	**3.58**	**3.67**	**3.77**
	3.90	4.09	4.27	4.44	4.60	4.76	4.91	5.06	5.21	5.35	5.49
	1.46	1.53	1.60	1.66	1.72	1.78	1.84	1.89	1.95	2.00	2.05
RCA 2	**2.15**	**2.25**	**2.35**	**2.44**	**2.53**	**2.62**	**2.70**	**2.78**	**2.86**	**2.94**	**3.01**
	3.17	3.32	3.46	3.60	3.73	3.85	3.98	4.10	4.21	4.32	4.43
	2.49	2.62	2.75	2.87	2.98	3.09	3.20	3.31	3.41	3.51	3.61
Distance to bifurcation	**4.93**	**5.19**	**5.44**	**5.68**	**5.91**	**6.13**	**6.35**	**6.56**	**6.76**	**6.96**	**7.16**
	9.78	10.29	10.78	11.26	11.71	12.15	12.58	13.00	13.41	13.80	14.19

The estimated values are in bold, the values above are −2SD, and the values below are +2SD. LMCA = left main coronary artery; prox. orig. = proximal origin, LAD = left anterior descending artery, CX = circumflex, RCA = right coronary artery.

## Data Availability

The data presented in this study are available on request from the corresponding author. The data are not publicly available due to privacy issue.

## Supplementary Materials

The following supporting information can be downloaded at: https://www.mdpi.com/article/10.3390/diagnostics14101029/s1, Table S1: Inter and intra-observer analysis. Intraclass correlation coefficient and coefficient of variation.
