# Supplementary material for: Comprehensive Two-Dimensional Pediatric Echocardiographic Nomograms for Coronary Artery Sizes in Caucasian Children and Comparison among Major Nomograms
Source: Diagnostics (Basel). 2024 May 16;14(10):1029. doi: 10.3390/diagnostics14101029 (PMC11119586; doi:10.3390/diagnostics14101029)
Supplement: Supplementary file 1 [file diagnostics-14-01029-s001.zip › diagnostics-2981131-supplementary.pdf]

Supplemental Table S1. Inter and intra-observer analysis. Intraclass Correlation Coefficient and Coefficient of Variation.

Measurements	*ICC Inter-observer*	*p value Inter-observer*	*ICC Intra-observer*	*p value Intra-observer*	*CV Inter-observer*	*CV Intra-observer*
LMCA	0.893 (0.778-0.949)	<0.001	0.911 (0.833-0.941)	<0.001	5.1%	3.4%
RCA	0.917 (0.799-0.970)	<0.001	0.945 (0.868-0.978)	<0.001	9.5%	6.5%
LAD	0.902 (0.789-0.951)	<0.001	0.949 (0.894-0.973)	<0.001	4.8%	3.5%
Cx	0.932 (0.857-0.966)	<0.001	0.952 (0.927-0.963)	<0.001	10.7%	7.2%

ICC =*Intraclass* Correlation Coefficient; CV= Coefficient of Variation, LMCA=left main coronary artery; LAD=left anterior descending artery, CX=circumflex, RCA=right coronary artery
